# Predictive model of language deficit after removing glioma involving language areas under general anesthesia

**DOI:** 10.3389/fonc.2022.1090170

**Published:** 2023-01-19

**Authors:** Meng Cui, Qingbao Guo, Yihong Chi, Meng Zhang, Hui Yang, Xin Gao, Hewen Chen, Yukun Liu, Xiaodong Ma

**Affiliations:** ^1^ Department of Emergency, The Sixth Medical Center, Chinese People's Liberation Army General Hospital, Beijing, China; ^2^ Medical School of Chinese People's Liberation Army, Beijing, China; ^3^ Department of Neurosurgery, The First Medical Center, Chinese People's Liberation Army General Hospital, Beijing, China; ^4^ Department of Information Technology, Xian Janssen Pharmaceutical Ltd., Beijing, China; ^5^ Department of Neurosurgery, The Second Hospital of Southern District of Chinese People's Liberation Army Navy, Sanya, China

**Keywords:** glioma, language, multimodal techniques, general anesthesia, predictive model

## Abstract

**Purpose:**

To establish a predictive model to predict the occurrence of language deficit for patients after surgery of glioma involving language areas (GILAs) under general anesthesia (GA).

**Methods:**

Patients with GILAs were retrospectively collected in our center between January 2009 and December 2020. Clinical variables (age, sex, aphasia quotient [AQ], seizures and KPS), tumor-related variables (recurrent tumor or not, volume, language cortices invaded or not, shortest distance to language areas [SDLA], supplementary motor area or premotor area [SMA/PMA] involved or not and WHO grade) and intraoperative multimodal techniques (used or not) were analyzed by univariate and multivariate analysis to identify their association with temporary or permanent language deficits (TLD/PLD). The predictive model was established according to the identified significant variables. Receiver operating characteristic (ROC) curve was used to assess the accuracy of the predictive model.

**Results:**

Among 530 patients with GILAs, 498 patients and 441 patients were eligible to assess TLD and PLD respectively. The multimodal group had the higher EOR and rate of GTR than conventional group. The incidence of PLD was 13.4% in multimodal group, which was much lower than that (27.6%, P<0.001) in conventional group. Three factors were associated with TLD, including SDLA (OR=0.85, P<0.001), preoperative AQ (OR=1.04, P<0.001) and multimodal techniques used (OR=0.41, P<0.001). Four factors were associated with PLD, including SDLA (OR=0.83, P=0.001), SMA/PMA involved (OR=3.04, P=0.007), preoperative AQ (OR=1.03, P=0.002) and multimodal techniques used (OR=0.35, P<0.001). The optimal shortest distance thresholds in detecting the occurrence of TLD/PLD were 1.5 and 4mm respectively. The optimal AQ thresholds in detecting the occurrence of TLD/PLD were 52 and 61 respectively. The cutoff values of the predictive probability for TLD/PLD were 23.7% and 16.1%. The area under ROC curve of predictive models for TLD and PLD were 0.70 (95%CI: 0.65-0.75) and 0.72 (95%CI: 0.66-0.79) respectively.

**Conclusion:**

The use of multimodal techniques can reduce the risk of postoperative TLD/PLD after removing GILAs under general anesthesia. The established predictive model based on clinical variables can predict the probability of occurrence of TLD and PLD, and it had a moderate predictive accuracy.

## Introduction

Glioma is the most common primary intracranial tumor, with an annual incidence of 4.67-5.73 per 100,000 (1). With the introduction of molecular mechanisms into the WHO classification of glioma, the treatment of glioma is developing a multidisciplinary, individualized, functional and preventive comprehensive treatment strategy, including surgery, postoperative radiotherapy, chemotherapy, immunotherapy and tumor-treating field (TTF), which can improve the outcome and survival of patients with glioma (2–6). Surgery is still the core method of comprehensive treatment, and many studies have proven that increasing the extent of resection (EOR) can prolong survival against glioma (7, 8). However, more aggressive resection may cause injury to normal brain tissue, especially eloquent areas, and their injury will lead to neurological deficits postoperatively. Language is an important neurological function of human, so maximal safe resection is the goal when removing the gliomas involving language areas (GILAs). Thus, the language cortices and tracts should be protected well while the maximal EOR is achieved.

Although direct electrical stimulation (DES) under awake craniotomy is the method of gold standard in mapping language areas when removing GILAs, multimodal techniques (neuronavigation based on multimodal imaging, intraoperative MRI [iMRI] and intraoperative neuromonitoring [IONM]) make maximal safe resection of GILAs possible under general anesthesia (9). An increasing number of studies believe that resection assisted by multimodal techniques under general anesthesia can achieve similar outcomes to awake craniotomy for patients with GILAs and takes less time with lower intraoperative risk (10, 11). We began to use multimodal techniques to remove GILAs under general anesthesia since 2009, and this surgery approach was proved to be effective and safe in our previous studies (12–14). According to our previous experience, some pre-, intra- and postoperative factors may be associated with temporary or permanent language deficits (TLD or PLD). In this study, we aimed to identify significant factors associated with TLD and PLD. Then based on these factors, a predictive model can be established to predict the occurrence of TLD and PLD. This model is expected to help surgeon and patients make appropriate choice on intraoperative techniques.

## Methods

### Patient selection

Retrospective clinical data were reviewed from electronic medical records in the Department of Neurosurgery at our center between January 2009 and December 2020. This study was approved by our institutional ethics committee. Written informed consent was signed by all patients or their relatives before surgery. These data were treated with privacy and reviewed to identify GILAs according to the following inclusion criteria, 1) pathology confirmed as supratentorial glioma based on the WHO classification of tumors of the central nervous system (3); 2) patients older than 6 years who can be assessed for language completely; 3) the tumor within 2 cm of language areas (language cortices and/or tracts) on preoperative MRI; 4) resection under general anesthesia; 5) pre/postoperative language function assessment and follow-up were completed. The exclusion criteria were as follows: 1) infratentorial glioma; 2) <6 years old; 3) biopsy only; and 4) loss of pre/postoperative MRI data; 5) loss of follow-up.

### Patient grouping

The patients were divided into occurrence group (TLD/PLD occurred) and non-occurrence group (TLD/PLD did not occur). The patients were also divided into conventional group (neuronavigation alone), and multimodal group (combined use of neuronavigation, iMRI, with/without DES/IONM).

### Preoperative variables

Preoperative general variables included age, sex, left or right-hander, symptoms, aphasia quotient (AQ) by Western Aphasia Battery testing (AQ≥93.8 and <93.8 were defined as normal and aphasia, respectively) (15–17), seizures (divided into no seizures, drug-controlled seizures and drug intractable seizures) and KPS.

Tumor-related variables included location, recurrent tumor or not, volume (cm^3^), language cortices invaded or not, shortest distance to language cortices or tracts (mm), supplementary motor area or premotor area (SMA/PMA) involved or not, histopathology, WHO grade. If the tumor was near language area but did not invaded it directly, the nearest distance was from border of tumor to language area. If the tumor invaded it directly, the nearest distance was 0.

### Outcome variables

The outcome variables included EOR, postoperative 3-month/6-month AQ and KPS, other surgery-related complications (intracranial infection, hemorrhage, ischemia, severe brain edema, hydrocephalus and leakage of cerebrospinal fluid, etc.), seizures and their control, temporary and permanent language deficits, postoperative radiotherapy (or not), cycles of TMZ chemotherapy, progression-free survival (PFS) and overall survival (OS). Language deficit was defined as the deterioration of postoperative language function at different time points compared to preoperative functional status of patients. According to De Witt Hamer 2012, the time point of TLD was defined as within 3 months postoperatively (18). The time point of PLD ranged from 2 weeks to 6 months in previous studies. Because the language function still improved after 3 months, according the definition of some studies we defined the time point of PLD as >6 months postoperatively (19–21). Furthermore in order to avoid the situation of language deficit caused by recurrent tumor, the patients of PFS ≤ 3 months were excluded when counting the cases of TLD up, the patients of PFS ≤ 6 months were excluded when counting the cases of PLD up.

### Surgery process and language areas preservation

All patients underwent preoperative MRI on a 1.5-T scanner (Siemens Espree, Erlangen, Germany). During the preoperative BOLD-fMRI scanning, the patients were asked to perform tasks of “number counting”, “picture naming” and “word/sentence making”. So that the language cortices can be activated on MRI. The MRI data were imported into the Brainlab software, iPlan 3.0 was used to perform preoperative surgical plan. All delineations of the region of interest (ROI) were completed by a board-certified neuroradiologist with 8 years of experience and a surgeon. The ROIs included tumor and language areas. The delineation of language cortices was based on anatomy and activated regions of BOLD-fMRI; then according to these seed areas, the language tracts were reconstructed. We depicted Broca area, Wernicke area and arcuate fasciculus for all patients. Because these areas were associated with language most directly, the preservation of them was enough for most patients. Sometimes we also depicted the angular gyrus, supramarginal gyrus, ventral premotor cortex and reconstructed inferior occipito-frontal tract, frontal aslant tract, etc. The 3 dimensional images of tumor and language cortices and tracts can be reconstructed and presented on screen, so that the shortest geodesic distance to language areas (cortices or tracts) can be calculated by measuring tools of iPlan. Finally, the surgical plan data were imported into neuronavigation. We performed most surgeries assisted by neuronavigation, iMRI and DES/IONM (multimodal group). The use of multimodal techniques can guide the surgeon to remove tumor and preserve language areas in real time. Some patients were performed resection guided by neuronavigation alone (conventional group). The choice of multimodal techniques was based on the volume and location of tumor, difficulty of resection, possibility of intraoperative residual tumor and language damage, patients’ wishes and surgeon’s experience.

### Tumor measurements

Tumor volume were measured with iPlan in Brainlab (Feldkirchen, Germany). The EOR was defined as (preoperative tumor volume – postoperative residual tumor volume)/preoperative tumor volume × 100. Gross total resection (GTR) was defined as EOR=100% in this study.

### Postoperative treatment and follow-up

Patients with LGG were recommended to receive postoperative radiotherapy (60 Gy) and adjuvant TMZ chemotherapy (150-200 mg/m^2^/day). Patients with HGG were recommended to receive radiotherapy plus concomitant (60 Gy + TMZ 75 mg/m^2^/day) and adjuvant TMZ chemotherapy (150-200 mg/m^2^/day). Regular MRI scanning was performed for patients every 3 months. The patients were followed up by an outpatient service or telephone every 3 months, and the follow-up time was up to December 2021.

### Statistical analysis

SPSS 21.0 was used to perform the statistical analysis. The Shapiro–Wilk test was used to test the normality of the data, and Levene’s test was used to test the homogeneity of variance of the samples. The Student’s t and χ^2^ (or Fisher’s exact test) tests were used to compare continuous parametric and categorical variables between groups, respectively. The Mann–Whitney *U* test was used to compare continuous nonparametric variables. Logistic regression was used to perform univariate and multivariate analysis. The predictive model was as follows: ln (P/1-P) = β0+β1X1+β2X2+…+βmXm (P was the occurrence probability of language deficits, β0 was a constant, Xm was the value of the variable, and βm was the regression coefficient). The accuracy of model was assessed by receiver operating characteristic (ROC) curve. A P value <0.05 was considered statistically significant. The univariate analysis set P<0.1 as the significance level.

## Results

Among 682 GILAs, 530 patients were included finally. One hundred and fifty-two patients were excluded because of age (<6 years old), biopsy only, loss of MRI data, awake craniotomy or loss of follow-up. Among these included patients, 498 patients were eligible to assess TLD because their PFS were longer than 3 months, 441 patient were eligible to assess PLD because their PFS were longer than 6 months. Among the 530 patients, 363 (355 [97.8%] right-handers) were performed surgery assisted by multimodal techniques and 167 (162 [97.0%] right-handers) were performed surgery assisted by neuronavigation alone.

### Comparison between occurrence group and non-occurrence group

Four hundred and ninety-eight patients with GILAs were assessed for the occurrence of TLD. One hundred and sixteen patients had TLD and 382 did not have TLD within 3 months postoperatively. Clinical and tumor factors were compared between the occurrence group and non-occurrence group (**Supplementary Table 1**). Compared to non-occurrence group, the occurrence group had shorter median distance between tumor and language areas (0.47mm versus 2.11mm, P=0.03), higher median preoperative AQ (100 versus 91.3, P=0.001) and lower rate of using multimodal techniques (56.0% versus 74.1%, P<0.001).

Four hundred and forty-one patients with GILAs were assessed for the occurrence of PLD. Seventy-seven patients had PLD and 364 did not have PLD on 6 months postoperatively. The comparison results between the occurrence group and non-occurrence group were presented in **Supplementary Table 2**. Compared to non-occurrence group, the occurrence group had shorter median distance between tumor and language areas (0mm versus 2.26mm, P=0.01), higher preoperative AQ (P=0.02), higher rate of SMA/PMA involved (18.2% versus 8.5%, P=0.02) and lower rate of using multimodal techniques (54.5% versus 74.7%, P<0.001).

### Comparison between conventional group and multimodal group

The multimodal group had the higher median EOR and rate of GTR than conventional group. The incidence of PLD was 13.4% in multimodal group, which was much lower than that (27.6%, P<0.001) in conventional group. The multimodal group also had higher KPS than conventional group on 3 and 6 months postoperatively. The incidences of other complications and seizure were similar in both groups (**Table 1**).

**Table 1:** Comparison of outcomes between multimodal and conventional groups.

Variables	Multimodal group (N=363)	Conventional group (N=167)	P
EOR (%), median (IQR)	100 (98.43-100)	94.97 (90.14-100)	**<0.001**
GTR (EOR=100%)	264 (72.7)	71 (42.5)	**<0.001**
Other complications, N (%)	21 (5.8)	12 (7.2)	0.54
Seizures, N (%)	35 (9.6)	14 (8.4)	0.64
KPS, within 3 months, mean±sd	81.6 ± 15.0	77.9 ± 16.7	**0.02**
KPS, within 6 months, mean±sd	84.9 ± 14.6	82.1 ± 14.7	**0.04**
Temporary language deficit, N (%)	65 (18.7)	51 (34.0)	**<0.001**
Permanent language deficit, N (%)	42 (13.4)	35 (27.6)	**<0.001**

Boldface type indicated statistical significance.

### Factors associated with TLD

Univariate analysis showed that 4 factors were associated with the occurrence of TLD (P<0.1) (**Table 2**).

**Table 2:** Factors for temporary language deficit by univariate analysis.

Factors	OR (95%CI)	P
Sex (Male vs Female)	1.08 (0.71-1.66)	0.72
Age	1.00 (0.98-1.01)	0.52
Recurrent tumor vs primary tumor	0.68 (0.38-1.22)	0.19
WHO grade (HGG vs LGG)	0.95 (0.59-1.52)	0.82
Tumor volume	1.00 (1.00-1.01)	0.23
Tumor location (reference: Frontal/Frontal insular)		
Temporal/Temporal insular	0.94 (0.58-1.53)	0.81
Frontal temporal/Frontotemporal insular	0.84 (0.46-1.54)	0.57
Insular/Parietal/Parietal temporal/Parietooccipital/Other locations	0.58 (0.27-1.27)	0.18
Shortest distance to language areas	0.92 (0.85-0.99)	**0.03**
Language cortices involved vs not involved	0.85 (0.56-1.29)	0.45
SMA/PMA invaded vs not invaded	1.45 (0.75-2.82)	0.27
Preoperative AQ	1.03 (1.01-1.04)	**<0.001**
Preoperative seizure (Yes vs No)	1.05 (0.67-1.64)	0.83
Drug intractable seizures (Yes vs No)	1.16 (0.50-2.66)	0.73
Preoperative KPS	1.01 (0.99-1.03)	0.26
EOR	0.97 (0.96-0.99)	**0.002**
Other complications (Yes vs No)	1.52 (0.80-2.90)	0.20
Multimodal techniques (used vs not used)	0.45 (0.29-0.69)	**<0.001**

Boldface type indicated statistical significance.

The optimal shortest distance threshold was 1.5mm in differentiating TLD with no TLD, thus if the tumor located within 1.5mm of language areas, the patient tended to have postoperative TLD (**Table 3**). The optimal AQ threshold was 52 in differentiating TLD with no TLD, thus if the AQ was more than 52, the patient tended to have postoperative TLD (**Table 4**).

**Table 3:** Optimal shortest distance threshold in differentiating TLD with no TLD by reduction of 0.5mm.

Univariate analysis	Shortest distance (≤3mm)	Shortest distance (≤2mm)	Shortest distance (≤1.5mm)	Shortest distance (≤1mm)
OR for TLD (95%CI)	1.48 (0.97-2.26)	1.50 (0.98-2.29)	1.52 (1.00-2.32)	1.57 (1.04-2.39)
P value	0.07	0.06	**0.049**	**0.034**

Boldface type indicated statistical significance.

**Table 4:** Optimal AQ threshold in differentiating TLD with no TLD by increments of 1 AQ.

Univariate analysis	AQ (≥50)	AQ (≥51)	AQ (≥52)	AQ (≥53)	AQ (≥55)	AQ (≥60)
OR for TLD (95%CI)	7.37 (0.98-55.16)	7.37 (0.98-55.16)	8.40 (1.13-62.58)	9.10 (1.22-67.60)	11.60 (1.57-85.62)	13.08 (1.78-96.25)
P value	0.052	0.052	**0.038**	**0.031**	**0.016**	**0.012**

Boldface type indicated statistical significance.

Multivariate analysis indicated that 3 factors were associated with TLD significantly, including shortest distance to language areas (OR=0.85, P<0.001), preoperative AQ (OR=1.04, P<0.001) and multimodal techniques used (OR=0.41, P<0.001) (**Table 5**). The predictive model of the probability of TLD was ln (P/1-P) = -1.78 -0.16X1 +0.037X2 -0.884X4, and the range of P was [4.8%, 87.2%].

**Table 5:** Factors for temporary language deficit by multivariate analysis.

Factors	OR (95%CI)	P
Shortest distance to language areas (X1)	0.85 (0.78-0.93)	**<0.001**
Preoperative AQ (X2)	1.04 (1.02-1.06)	**<0.001**
EOR (X3)	0.98 (0.96-1.00)	0.06
Multimodal techniques (used vs not used) (X4)	0.41 (0.26-0.65)	**<0.001**

Boldface type indicated statistical significance.

**Figure 1:**
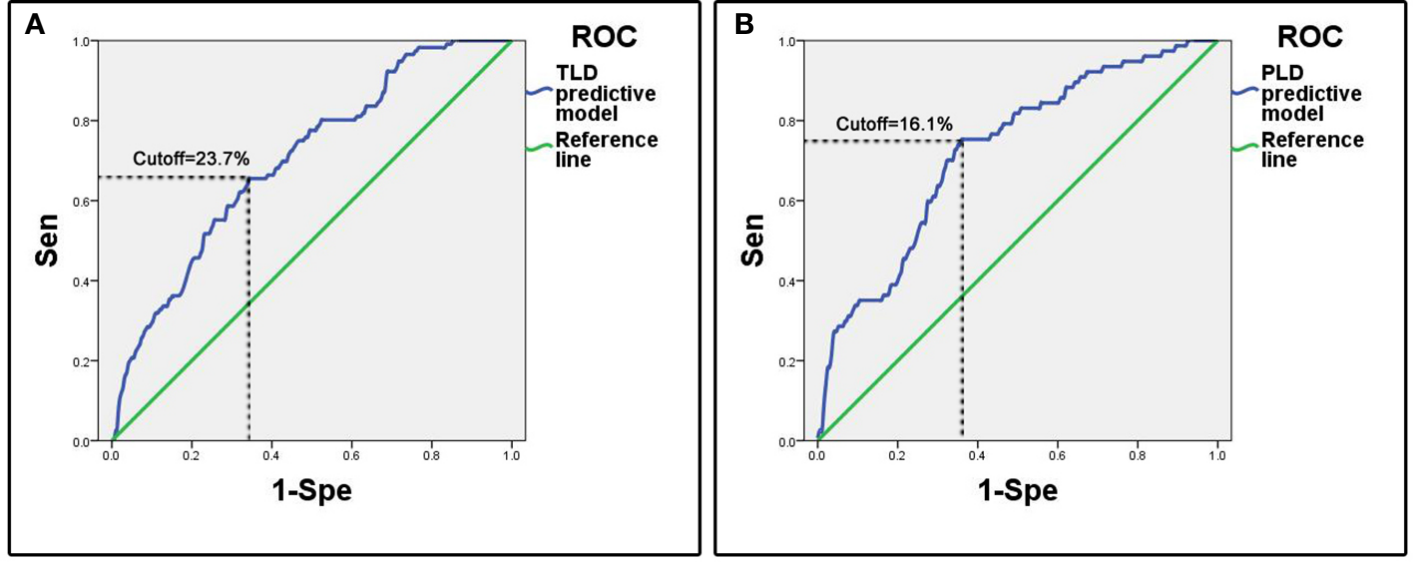
ROC curves of predictive models. **(A)** Predictive model of temporary language deficit, the cutoff value of predictive probability of TLD was 23.7%, the Sen was 0.66 and the Spe was 0.66. **(B)** Predictive model of permanent language deficit, the cutoff value of predictive probability of PLD was 16.1%, the Sen was 0.75 and the Spe was 0.64.

The ROC analysis showed that the area under curve (AUC) of ROC was 0.70 (95%CI: 0.65-0.75). The cutoff predictive probability of TLD was 23.7%. In this case the sensitivity (Sen) was 0.66, the specificity (Spe) was 0.66, the diagnostic odds ratio (DOR) was 3.64, the positive predictive value (+PV) was 0.37, and the negative predictive value (-PV) was 0.86 (**Figure 1**).

### Factors associated with PLD

Univariate analysis showed that 6 factors were associated with the occurrence of TLD (P<0.1) (**Table 6**).

**Table 6:** Factors for permanent language deficit by univariate analysis.

Factors	OR (95%CI)	P
Sex (Male vs Female)	1.10 (0.66-1.82)	0.72
Age	1.00 (0.98-1.02)	0.70
Recurrent tumor vs primary tumor	0.75 (0.38-1.50)	0.42
WHO grade (HGG vs LGG)	1.17 (0.67-2.04)	0.58
Tumor volume	1.00 (1.00-1.01)	0.76
Tumor location (reference: Frontal/Frontal insular)		
Temporal/Temporal insular	0.69 (0.39-1.24)	0.22
Frontal temporal/Frontotemporal insular	0.61 (0.29-1.26)	0.57
Insular/Parietal/Parietal temporal/Parietooccipital/Other locations	0.39 (0.15-1.05)	**0.06**
Shortest distance to language areas	0.91 (0.84-1.00)	**0.05**
Language cortices involved vs not involved	1.01 (0.62-1.66)	0.96
SMA/PMA invaded vs not invaded	2.39 (1.20-4.74)	**0.01**
Preoperative AQ	1.02 (1.00-1.04)	**0.02**
Preoperative seizure (Yes vs No)	1.41 (0.85-2.33)	0.18
Drug intractable seizures (Yes vs No)	1.20 (0.47-3.04)	0.71
Preoperative KPS	1.01 (1.00-1.03)	0.24
EOR	0.98 (0.96-1.00)	**0.07**
Complications (Yes vs No)	0.65 (0.25-1.72)	0.39
Multimodal techniques (used vs not used)	0.41 (0.24-0.67)	**<0.001**

Boldface type indicated statistical significance.

The optimal shortest distance threshold was 4mm in differentiating PLD with no PLD, thus if the tumor located within 4mm of language areas, the patient tended to have postoperative PLD (**Table 7**). The optimal AQ threshold was 61 in differentiating PLD with no PLD, thus if the AQ was more than 61, the patient tended to have postoperative PLD (**Table 8**).

**Table 7:** Optimal shortest distance threshold in differentiating PLD with no PLD by reduction of 0.5mm.

Univariate analysis	Shortest distance (≤5mm)	Shortest distance (≤4.5mm)	Shortest distance (≤4mm)	Shortest distance (≤3mm)
OR for PLD (95%CI)	1.41 (0.72-2.74)	1.84 (0.97-3.49)	1.83 (1.01-3.31)	1.78 (1.05-3.03)
P value	0.32	0.06	**0.046**	**0.033**

Boldface type indicated statistical significance.

**Table 8:** Optimal AQ threshold in differentiating PLD with no PLD by increments of 1 AQ.

Univariate analysis	AQ (≥55)	AQ (≥60)	AQ (≥61)	AQ (≥62)	AQ (≥63)	AQ (≥65)
OR for PLD (95%CI)	6.58 (0.88-49.05)	7.33 (0.99-54.45)	7.83 (1.06-58.10)	8.60 (1.16-63.66)	9.12 (1.23-67.43)	2.91 (1.02-8.30)
P value	0.07	0.052	**0.044**	**0.035**	**0.03**	**0.046**

Boldface type indicated statistical significance.

Multivariate analysis indicated 4 factors were associated with PLD significantly, including shortest distance to language areas (OR=0.83, P=0.001), SMA/PMA involved (OR=3.04, P=0.007), preoperative AQ (OR=1.03, P=0.002) and multimodal techniques used (OR=0.35, P<0.001) (**Table 9**). The predictive model of the probability of PLD was ln (P/1-P) = -2.098 -0.186X2 +1.112X3 +0.032X4 -1.046X6, and the range of P was [0.1%, 90.2%].

**Table 9:** Factors for permanent language deficit by multivariate analysis.

Factors	OR (95%CI)	P	
Tumor location (X1, reference: Frontal/Frontal insular)			
Temporal/Temporal insular	0.84 (0.43-1.62)	0.60
Frontal temporal/Frontotemporal insular	0.75 (0.33-1.69)	0.49
Insular/Parietal/Parietal temporal/Parietooccipital/Other locations	0.53 (0.19-1.51)	0.23
Shortest distance to language areas (X2)	0.83 (0.75-0.93)	**0.001**	
SMA/PMA invaded vs not invaded (X3)	3.04 (1.35-6.84)	**0.007**	
Preoperative AQ (X4)	1.03 (1.01-1.05)	**0.002**	
EOR (X5)	0.99 (0.96-1.01)	0.33	
Multimodal techniques (used vs not used) (X6)	0.35 (0.20-0.61)	**<0.001**	

Boldface type indicated statistical significance.

The ROC analysis showed that the AUC of ROC was 0.72 (95%CI: 0.66-0.79). The cutoff of predictive probability of PLD was 16.1%. In this case the Sen was 0.75, the Spe was 0.64, the DOR was 5.49, the positive +PV was 0.31, and the -PV was 0.92 (**Figure 1**).

## Discussion

Maximal safe resection is the goal of surgery of glioma involving eloquent areas. The preservation of language is an important factor that should be considered in the resection of GILAs in the dominant hemisphere. DES combined with awake craniotomy has been the gold standard method for the surgery of GILAs and it continuously develops and innovates (22). Many methods have been developed to increase the accuracy of MRI in functional area localization, for example, a combination of seed-based correlation mapping and spatially independent component analysis, a combination of tb-fMRI and rs-MRI, and optimization of the DTI tract reconstruction algorithm. Furthermore, various intraoperative imaging techniques, especially intraoperative MRI (iMRI), can overcome brain drift defects and increase EOR. Therefore, an increasing number of studies have indicated that neuronavigation based on multimodal imaging, iMRI, DES and IONM (multimodal techniques) can achieve maximal safe resection of GILAs under general anesthesia (23, 24). Our center began to use neuronavigation in glioma surgery in 2002 and has used intraoperative multimodal techniques since 2009. Previous studies reported the incidences of PLD ranged from 0 to 14.8% when removing GILAs under awake craniotomy, the incidence was around 8% in most studies. Although our multimodal group of general anesthesia had a little higher incidence of PLD (13.4%) than previous studies of awake craniotomy, we achieved much higher rate of GTR (72.7%) than all previous studies of awake craniotomy (ranged from 25.5% to 72.0%, most around 50%) (24–36). Thus when the patients were underwent surgery assisted by multimodal techniques under general anesthesia, their outcome was not inferior to those under awake craniotomy. However many factors may also cause temporary or permanent language deficits after surgery under general anesthesia. What factors are associated with TLD or PLD remains unclear. In this study, we tried to identify significant factors of TLD and PLD based on our large cohort of patients.

Shortest distance to language areas was both associated with TLD (OR=0.85) and PLD (OR=0.83), which indicated that the shorter the distance between tumor and language areas was, the higher probability of occurrence of TLD and PLD was. This phenomenon was obvious to be explained, if the tumor was close to language areas, it may more likely invade and destroy the language function. Meanwhile preoperative AQ was both associated with TLD (OR=1.04) and PLD (OR=1.03), which demonstrated that a higher AQ of the patient meant a higher probability of occurrence of TLD and PLD. This result can be explained by the more obvious change effect of language function tested by us in patients with higher AQ. Optimal shortest distance threshold for detecting TLD was 1.5mm and for detecting PLD was 4mm. Optimal AQ threshold for detecting TLD was 52 and for detecting PLD was 61. This result indicates that if the border of tumor is less than 4mm from language areas and the AQ is more than 61, the patients tend to have the higher probability of occurrence of PLD. In this case, multimodal techniques should be suggested to be used, in addition awake craniotomy combined with DES can preserve the language function more effectively for this kind of patients (31). The iMRI can both benefit the preservation of temporary and permanent language function. The iMRI was a valuable tool to correct brain drift by updating neuronavigation and reconstructing language cortices and tracts. It can also detect residual tumor and update surgical plan, the further resection can increase the EOR significantly (37, 38). So for all patients with GILAs, iMRI should be suggested.

Tumor location was associated with PLD in univariate analysis. Compared with the tumors of frontal/frontal insular lobes, the tumors of insular and parietal lobes were associated with a lower probability of PLD by univariate analysis (OR=0.39, P=0.06). As we know, frontal lobe, especially inferior frontal gyrus and its related tracts played a main role in language. While some part of insular and parietal lobes also participated in language function, for example, angular gyrus and supramarginal gyrus were located in inferior parietal lobe, the insular lobe had efferent and afferent connections with temporal lobe (39, 40). Our result indicated that gliomas of frontal lobe may have more impact on postoperative language function, which can cause more PLD of patients. However this association was not significant in multivariate analysis. We considered it was because the use of intraoperative multimodal techniques for many patients reduced the influence of tumor location on PLD, and tumor location was not more important than other factors.

Many previous studies had proved the influence of SMA and PMA on language function. Language function was considered controlled by a network that involving many cortical and subcortical areas. SMA and PMA were two important areas that took part in language function. Hertrich 2016 reported that SMA was associated with both speech motor control and language processing (41). Bathla 2019 reported that the central SMA can participate in the connectivity with both motor and language networks (42). Van Geemen 2014 reported that the ventral PMA played a role in language production and fluency and its plasticity was limited (43). Another study Duffau, 2004 also proved the relevance between language function and ventral PMA (including its descending subcortical tract) (44). So our result supported the importance of SMA/PMA on language function of previous studies. Our result showed that SMA or PMA involved was associated with the occurrence of PLD, but was not associated with the occurrence of TLD. This phenomenon may be explained by the long-term effects of destroyed SMA/PMA. The AQ test may not have the ability to detect the subtle change of advanced language in a short-term, so the AQ may not change a lot, which caused no association between SMA/PMA involved and TLD. This phenomenon should be testified by studies in future.

To the authors’ knowledge, this study is the largest series utilizing a multimodal approach guiding GILAs resection under general anesthesia. We established predictive model based on clinical factors and identified the cutoff values of predictive probability for TLD and PLD respectively. If a patient has a high preoperative AQ and the tumor is close to language areas and involves SMA/PMA, the predictive probability for TLD/PLD is higher than the cutoff, he/she will have a high predictive probability of TLD/PLD. In this case, we should use multimodal techniques (especially iMRI) under general anesthesia to preserve language more effectively. In addition, awake craniotomy combined with DES can be used for patients having a very high predictive probability of TLD/PLD. Although our predictive model had a moderate accuracy, it can still guide surgeon and patients of GILAs to choose an appropriate surgery strategy and avoid unnecessary techniques used. Furthermore, the model for TLD and PLD both had the high -PV (0.86 and 0.92) and low +PV (0.37 and 0.31). Thus, if a patient had a calculated probability less than cutoff value, resection under general anesthesia can ensure a low incidence of TLD/PLD. So this model can provide surgeon and patients a reference of incidence of TLD/PLD to help them make decision of what intraoperative techniques to be used.

Some limitations existed in this study. (1) Retrospective studies have inherent limitations that may cause selection bias, recall bias and loss to follow-up bias. (2) In order to facilitate data analysis, tumor location cannot be classified too many categories, we only classified 4 main categories in our cohort. But the language network was complex, gliomas involving different language areas can cause different types of language deficits. In further study, the tumor location should be divided in detail to explore its association with postoperative language function of GILAs. (3) The predictive model was based on cohort of patients under general anesthesia, so it was only applicable to patients underwent surgery under general anesthesia. The model should be validated with further prospective studies. Awake craniotomy should be also added as a factor that associated with language deficit to improve this model in future, which will make this model have a wider applied range.

## Conclusion

The use of multimodal techniques can reduce the risk of postoperative TLD/PLD after removing GILAs under general anesthesia. The established predictive model indicated that higher AQ, shorter distance to language areas, SMA/PMA invaded and multimodal techniques not used were associated with the higher probability of occurrence of language deficit after resection of GILAs under general anesthesia. The optimal AQ threshold and shortest distance threshold in detecting TLD/PLD were also identified. The model had a moderate accuracy in predicting the occurrence of TLD/PLD. We can provide the surgeon and patients a guide to choose appropriate surgery strategy and techniques. The model should be validated with further prospective studies.

## Data availability statement

The raw data supporting the conclusions of this article will be made available by the authors, without undue reservation.

## Ethics statement

Retrospective clinical data were reviewed from electronic medical records in the Department of Neurosurgery at our center between January 2009 and December 2020. This study was approved by our institutional ethics committee. Written informed consent was signed by all patients or their relatives before surgery.

## Author contributions

MC: conceptualization, data collection, data analysis, data visualization, literature review, and manuscript writing and editing; QG, YC: data collection, data analysis, data visualization; MZ, HY, XG, HC, YL: data collection, data curation, reviewed speech and language. XM: conceptualization, methodology, supervision, and manuscript reviewing. All authors contributed to the article and approved the submitted version.
