# Supplementary material for: Predictive model of language deficit after removing glioma involving language areas under general anesthesia
Source: Front Oncol. 2023 Jan 19;12:1090170. doi: 10.3389/fonc.2022.1090170 (PMC9892894; doi:10.3389/fonc.2022.1090170)
Supplement: Supplementary file 1 [file Table_1.docx]

Supplementary table 1. Comparing variables between groups divided according to the occurrence of TLD or not

| **Factors** | **Occurrence group (N=116)** | **Non-occurrence group (N=382)** | **P** | |
| --- | --- | --- | --- | --- |
| Female, N (%) | 44 (37.9) | 152 (39.8) | 0.72 |  |
| Age | 46.1±15.3 | 47.0±13.2 | 0.55 |  |
| Recurrent tumor, N (%) | 16 (13.8) | 73 (19.1) | 0.19 |  |
| Right-handed, N (%) | 112 (96.6) | 373 (97.6) | 0.75 |  |
| LGG, N (%) | 31 (26.7) | 98 (25.7) | 0.82 |  |
| Tumor volume, median (IQR) | 48.31 (30.05-76.61) | 43.49 (26.85-68.50) | 0.13 |  |
| Tumor location, N (%) |  |  | 0.57 |  |
| Frontal/Frontal insular | 53 (45.7) | 158 (41.4) |  |  |
| Temporal/Temporal insular | 36 (31.0) | 114 (29.8) |  |  |
| Frontal temporal/Frontotemporal insular | 18 (15.5) | 64 (16.8) |  |  |
| Insular/Parietal/Parietal temporal/Parietooccipital/Other locations | 9 (7.8) | 46 (12.0) |  |  |
| Shortest distance to language areas, median (IQR) | 0.47 (0-3.74) | 2.11 (0-4.61) | **0.03** |  |
| Language cortices involved, N (%) | 50 (43.1) | 180 (47.1) | 0.45 |  |
| Involved SMA/PMA, N (%) | 14 (12.1) | 33 (8.6) | 0.27 |  |
| Preoperative AQ, median (IQR) | 100 (82.3-100) | 91.3 (74.0-100) | **0.001** |  |
| Preoperative seizure, N (%) | 38 (32.8) | 121 (31.7) | 0.83 |  |
| Drug intractable seizures, N (%) | 8 (6.9) | 23 (6.0) | 0.73 |  |
| Preoperative KPS, median (IQR) | 80 (60-87.5) | 70 (70-80) | 0.21 |  |
| Multimodal techniques, N (%) | 65 (56.0) | 283 (74.1) | **<0.001** |  |
