# Supplementary material for: Predictive model of language deficit after removing glioma involving language areas under general anesthesia
Source: Front Oncol. 2023 Jan 19;12:1090170. doi: 10.3389/fonc.2022.1090170 (PMC9892894; doi:10.3389/fonc.2022.1090170)
Supplement: Supplementary file 2 [file Table_2.docx]

Supplementary table 2. Comparing variables between groups divided according to the occurrence of PLD or not

| **Factors** | **Occurrence group (N=77)** | **Non-occurrence group (N=364)** | **P** | |
| --- | --- | --- | --- | --- |
| Female, N (%) | 29 (37.7) | 145 (39.8) | 0.72 |  |
| Age | 45.6±13.6 | 46.2±13.3 | 0.70 |  |
| Recurrent tumor, N (%) | 11 (14.3) | 66 (18.1) | 0.42 |  |
| Right-handed, N (%) | 74 (96.1) | 358 (98.4) | 0.41 |  |
| LGG, N (%) | 20 (26.0) | 106 (29.1) | 0.58 |  |
| Tumor volume, median (IQR) | 44.80 (29.48-69.66) | 44.73 (26.87-69.73) | 0.56 |  |
| Tumor location, N (%) |  |  | 0.17 |  |
| Frontal/Frontal insular | 40 (51.9) | 144 (39.6) |  |  |
| Temporal/Temporal insular | 21 (27.3) | 109 (29.9) |  |  |
| Frontal temporal/Frontotemporal insular | 11 (14.3) | 65 (17.9) |  |  |
| Insular/ Parietal/Parietal temporal/Parietooccipital/Other locations | 5 (6.5) | 46 (12.6) |  |  |
| Shortest distance to language areas, median (IQR) | 0 (0-3.53) | 2.26 (0-4.64) | **0.01** |  |
| Language cortices involved, N (%) | 36 (46.8) | 169 (46.4) | 0.96 |  |
| Involved SMA/PMA, N (%) | 14 (18.2) | 31 (8.5) | **0.02** |  |
| Preoperative AQ, median (IQR) | 100 (82.7-100) | 100 (75.3-100) | **0.02** |  |
| Preoperative seizure, N (%) | 32 (41.6) | 122 (33.5) | 0.18 |  |
| Drug intractable seizures, N (%) | 6 (7.8) | 24 (6.6) | 0.70 |  |
| Preoperative KPS, median (IQR) | 80 (70-80) | 80 (70-80) | 0.23 |  |
| Multimodal techniques, N (%) | 42 (54.5) | 272 (74.7) | **<0.001** |  |
